# An Overview of PDE4 Inhibitors in Clinical Trials: 2010 to Early 2022

**DOI:** 10.3390/molecules27154964

**Published:** 2022-08-04

**Authors:** Letizia Crocetti, Giuseppe Floresta, Agostino Cilibrizzi, Maria Paola Giovannoni

**Affiliations:** 1 NEUROFARBA, Pharmaceutical and Nutraceutical Section, University of Florence, Via Ugo Schiff 6, 50019 Sesto Fiorentino, Italy; 2 Department of Drug and Health Sciences, University of Catania, Viale A. Doria 6, 95125 Catania, Italy; 3 Institute of Pharmaceutical Science, King’s College London, Stamford Street, London SE1 9NH, UK

**Keywords:** phosphodiesterase 4, PDE4 inhibitors, clinical trials, respiratory diseases, skin diseases, rheumatoid arthritis, neurological disorders, COVID-19

## Abstract

Since the early 1980s, phosphodiesterase 4 (PDE4) has been an attractive target for the treatment of inflammation-based diseases. Several scientific advancements, by both academia and pharmaceutical companies, have enabled the identification of many synthetic ligands for this target, along with the acquisition of precise information on biological requirements and linked therapeutic opportunities. The transition from pre-clinical to clinical phase was not easy for the majority of these compounds, mainly due to their significant side effects, and it took almost thirty years for a PDE4 inhibitor to become a drug i.e., Roflumilast, used in the clinics for the treatment of chronic obstructive pulmonary disease. Since then, three additional compounds have reached the market a few years later: Crisaborole for atopic dermatitis, Apremilast for psoriatic arthritis and Ibudilast for Krabbe disease. The aim of this review is to provide an overview of the compounds that have reached clinical trials in the last ten years, with a focus on those most recently developed for respiratory, skin and neurological disorders.

## 1. Introduction

Starting from the discovery of phosphodiesterases (PDEs) by Sutherland and Rall in 1958 [1], there has been a continuous and wide interest by the medicinal chemistry community on the modulation of their activity. PDEs catalyse the hydrolysis of the phosphodiester bond of c-AMP and c-GMP affording the corresponding AMP and GMP inactive counterparts. In fact, the inhibition of PDE leads to an increase in cyclic nucleotide levels, which in turn play a prominent role as second messengers, in the regulation of a variety of cell functions, such as secretion, contraction, metabolism and growth [2,3,4,5].

**Figure 1:**
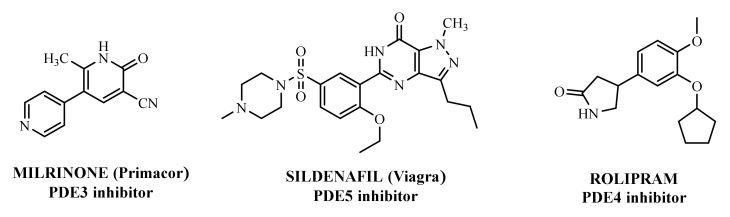
PDEs inhibitors.

The first important synthetic effort made during the ‘1980s by the pharmaceutical industry in the area of PDE led to the development and marketing by Sanofi-Aventis of the PDE3 inhibitor Milrinone [6,7] for the treatment of heart failure (Figure 1). However, this drug had many drawbacks and was later withdrawn from the market because of serious side effects in the long term. Indeed, chronic administration of Milrinone resulted in reduced survival, showing that chronic elevation of c-AMP in cardiac myocytes is associated with side effects provoking a major risk of fatal arrhythmias [8]. Milrinone is currently marketed exclusively for hospital use in cases of cardiac shock [9] and given its power, it is found in numerous clinical trials for pulmonary hypertension, septic shock and other (ClinicalTrials.gov Identifier: NCT04484675: Comparative Study Between Inhaled and Intravenous Milrinone in Patients With Severe Pulmonary Hypertension Undergoing Cardiac Surgery; ClinicalTrials.gov Identifier: NCT05122884: for the treatment/prevention of severe sepsis/septic shock, whose relatively common complication is myocardial dysfunction; ClinicalTrials.gov Identifier: NCT04362527: for the therapy of subarachnoid hemorrhage).

Another significant success was the launch of Sildenafil (Figure 1), the first oral PDE5 inhibitor originally studied for angina, and then approved by FDA and marketed by Pfizer as Viagra^®^ in 1998 for the treatment of male erectile dysfunction [10,11,12]. About seven years later it was also approved for pulmonary arterial hypertension and classified as an orphan drug by EMEA [13]. The commercialization of Sildenafil was quickly followed by the marketing of additional PDE5 inhibitors [14].

This remarkable achievement reinforced the assumption to intensify the investigations also in the field of PDE4 inhibitors, already widely explored as potential candidates for the treatment of chronic inflammatory disorders. In the development of these drugs, the most ambitious goal was to be able to limit the side effects, in order to overcome the difficulties found with PDE3 inhibitors, therefore allowing chronic use.

The interest for PDE4 by both academia and pharmaceutical companies is widely documented by the numerous reviews and patents published over the past twenty years [15,16,17,18,19,20,21,22,23,24,25,26] and despite the disappointing results obtained in clinical studies with various PDE4 inhibitors, as well as the early termination of trials on compounds under development, very important results have been obtained in this field, as highlighted by the PDE4 inhibitors currently in clinical use.

## 2. Overview of PDE4

The superfamily of PDEs is currently subclassified into 11 families, namely PDE1-PDE11, which are characterized by different kinetic properties, tissue distribution, responsiveness to endogenous regulators (Ca^2+^, calmodulin, c-GMP) and co-factors (Mg^2+^, Zn^2+^), sensitivity to synthetic inhibitors and, in some cases, substrate specificity (c-AMP or c-GMP) [27]. Each family is expressed by one or more genes; furthermore, alternative mRNA processing is responsible for the production of multiple splice variants. Thus, until now, more than 50 distinct human PDE proteins have been identified. The generally accepted nomenclature for the different gene products is based on two capital letters indicating the species (HS, Homo sapiens, RT Ratus norvegicus), followed by PDE; then there is an Arabic numeral indicating the family, which is followed, in turn, by a capital letter for the gene (A, B, C, D) and finally by an Arabic numeral for the splice variant [28,29].

**Figure 2:**
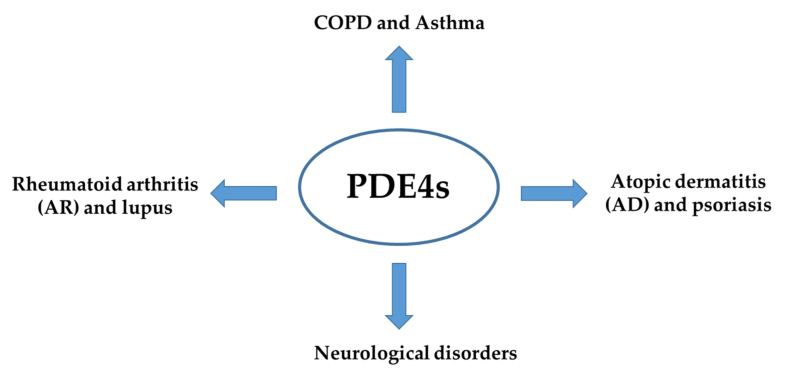
PDE4s involvement in the physio-pathogenesis of inflammatory diseases.

Phosphodiesterase-4 (PDE4) is the most diversified sub-family of phosphodiesterase (PDEs) and is abundantly expressed in a variety of cell types [30]. These enzymes have been found to mediate several physiological processes, such as brain functions, macrophage and monocyte activation, myocardial contractility, vascular smooth muscle proliferation and neutrophil infiltration, to name a few. Moreover, PDE4 has been reported to participate in the physio-pathogenesis of many inflammatory diseases such as rheumatoid arthritis, chronic obstructive pulmonary disease (COPD) and asthma (Figure 2). Additionally, PDE4 have shown roles in the progress and development of autoimmune diseases, cardiovascular diseases, and cancers [31,32].

Four different subtypes of PDE4 have been identified to date, namely PDE4A, PDE4B, PDE4C and PDE4D. The genes of these subtypes are located on chromosomes 19p13.2, 1p31, 19p13.11, and 5q12, and each of these subtypes can express from 3 to 11 different proteins, resulting in at least 25 different isoforms of PDE4 distributed within different cellular compartments and with different levels of expression. All the PDE4 have been reported to exist in three different forms based on their size i.e., long, short and super-short. The longer version of the protein has two conserved domains, UCR1 (of approximately 60 amino acids) and UCR2 (of approximately 80 amino acids) in the N-terminal region. Differently, the short form has only the UCR2 domain in full, while the super-short version of the protein has a truncated UCR2. At the C-terminus, all the PDE4 have a catalytic domain of 300–350 amino acids. The active site of the enzyme can be divided into three sections as shown by X-ray structures: (i) a pocket that interacts with the phosphate moiety of cAMP, (ii) two pockets that form interactions with small molecules inhibitors, and (iii) a solvated pocket [33,34]. Unfortunately, the high conserved sequence identity among the family of PDE4 make the discovery of isoform-selective inhibitors challenging.

The UCRs motifs of the PDE4 are also important for the PDE4 regulation [35]. PDE4 is regulated by transcriptional regulation (long-term) or post-translational modifications (short-term). When the protein kinase A (PKA) phosphorylates in a conserved PKA phosphorylation site the UCRs, it has been reported to regulate the PDE4 dimerization and catalytic activities [25]. In long-term regulation of PDE4, c-AMP concentration is increased and the activation of adenylyl cyclase (AC) by hormone mediate stimulation is assisted as a result of increased gene expression. Differently, in the short-term regulation, the activation of PKA is determined by the increased concentration of c-AMP levels. In turn, PKA phosphorylates specific serine residues in UCR1 of PDE4, producing a rapid increase in its activity. However, PDE4 activity is also regulated by other proteins such as Src family tyrosine protein kinases, arrestin and the receptor for activated C kinase 1 (RACK1) [35]. 

## 3. Inflammation and PDE4

**Figure 3:**
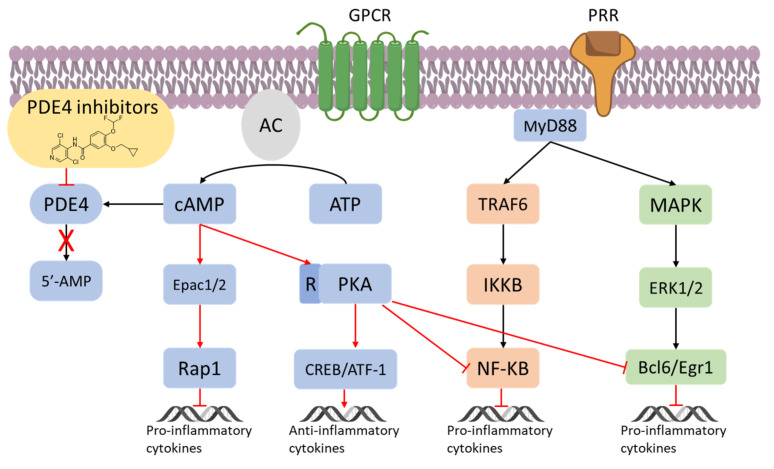
PDE4 inhibitors in the regulation of inflammatory responses [36,37,38].

The Nuclear Factor κ-light-chain-enhancer of activated B cells (NF-κB) can mediate cell-specific responses and pharmacological attempt to block its activation is being considered a new therapeutic option in inflammatory conditions (Figure 3) [36,37,38]. It is known that c-AMP interferes with the NF-κB signalling, being in parallel recognized as an immunosuppressive and anti-inflammatory actor as well [39]. As a result, PDE4 could be potentially useful to indirectly leverage the inactivation/activation of the NF-κB signalling in inflammation. For instance, in endothelial cells, the high levels of c-AMP produced by an adenylate cyclase activator (e.g., forskolin) have been reported to prevent the NF-κB-dependent gene transcription [40]. When macrophages are treated with ethanol chronically, the inhibition of PDE4 has been proved to decrease the TNF-α mRNA expression, by the intervention of the transcriptional modulation of NF-κB [41]. Moreover, the inhibition of PDE4 leads not only to less NF-κB-mediated TNF-α expression but also to activation of the PKA and increased synthesis of IL-10 [42]. Consequently, inhibitors of PDE4 may be useful to modulate, negatively or positively, gene expression. Variation in the NF-κB by PDE4 and c-AMP has also been reported in T cells, where PDE4 has been found to control the proliferation of T lymphocytes, along with the concentration of TNF-α and other interleukins such as IL-2, IL-4, and IL-5 [43]. The c-AMP has also been reported as a crucial mediator of the regulation of T cell suppression, by crossing the cell membrane of responder T cells, such as CD4-positive [44] and TH2 subsets [45], as well as inhibiting T cell proliferation. In the context of inflammation, PDE4 are known to promote also chemotaxis and degranulation in both eosinophils and neutrophils. These effects are mediated by the increased concentration of IL-8, leukotriene B4 and superoxide anion stimulated by PDE4 in neutrophils. Moreover, PDE4 has been reported to control the expression of adhesion molecules, such as the β2-integrin Mac-1 in neutrophils, resulting in augmented adhesion to vascular endothelial cells [46,47].

## 4. From In Vitro and Preclinical Profile of The First PDE 4 Inhibitors to Approved Drugs

In the context of PDE4 inhibitors, it would appear definitely appropriate to start with Rolipram (Figure 1), the synthesis of which dates back to 1977 [48,49]. Rolipram has certainly been the most investigated PDE4 inhibitor, from the first studies carried out shortly after its synthesis, to investigations conducted at present time [50,51,52,53,54,55,56], including further clinical trials (as detailed below). Already at the time of the first studies on Rolipram and its analogues, there was a body of experimental evidence (confirmed later on) that PDE4 inhibitors were able to suppress inflammatory and immunomodulatory responses in a variety of murine and human cells, to block superoxide generation in monocytes, macrophages, neutrophils and eosinophils, to reduce TNF-α release in monocytes and macrophages, and to suppress chemotaxis and phagocytosis [57,58]. It is important to highlight that in eosinophils, which are the effectors “par excellence” of asthma, PDE4 inhibitors can suppress superoxide generation, chemotaxis, degranulation, LTC4 synthesis and CD11b expression. In vivo, PDE4 inhibitors demonstrated bronchodilatory effects and the ability to reverse bronchospasm induced by a variety of agents, thus, the profile of selective PDE4 inhibitors appeared to fulfil the requirement for the treatment of inflammation-based pathologies such as asthma and chronic obstructive pulmonary disease (COPD) and some auto-immune diseases [15,59,60,61,62].

Even though such promising in vitro and preclinical results generated a large consensus on the concept of PDE4 as a valid target for the treatment of the above diseases, clinical evaluation of several potent and selective PDE4 inhibitors was strongly disappointing [63]. A lack of correlation between preclinical and clinical data was a prominent limitation. In this context, it is worth mentioning that the first clinical studies were performed with the first generation of PDE4 inhibitors (rolipram and congeners), which were characterized by serious adverse reactions found, at least in part, associated with the affinity for the high-affinity Rolipram binding site (HARBS) [64]. Only in 1996, it become evident that a better side-effect profile could be obtained with agents preferentially targeting the catalytic site over HARBS [65]. Then, this type of selectivity was not pursued any longer, and another type of selectivity versus the gene products A-D was proposed after these subtypes were characterized, their tissue distribution demonstrated and their different functional role suggested [66]. In the early 2000s, the PDE4D isoform was indicated as being responsible for PDE4 inhibitor-induced emesis [67], although recent studies suggest that it may not be the only factor involved in this side effect [68]; on the other hand, it has been demonstrated that PDE4B selective inhibitors produce potent anti-inflammatory and reduced emetic effects [69].

**Figure 4:**
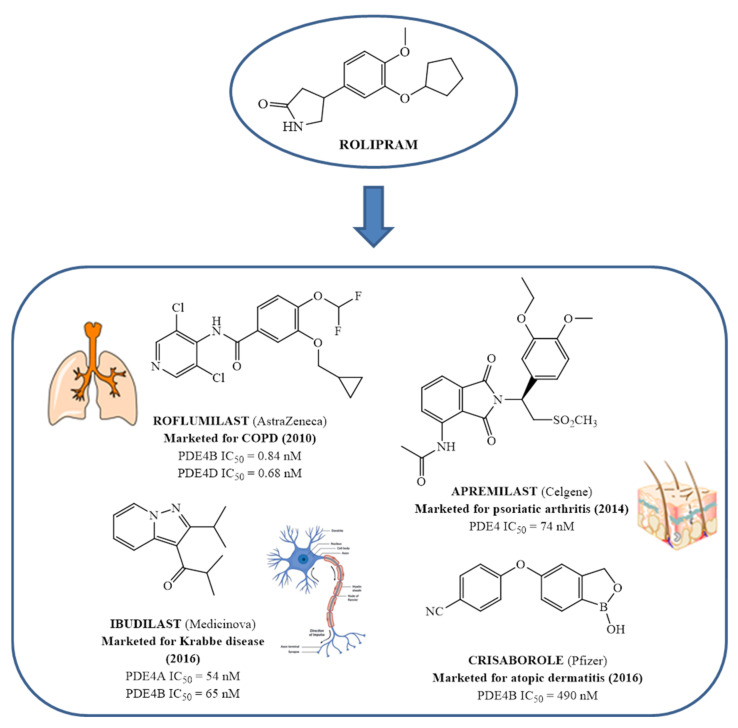
From Rolipram to marketed PDE4 inhibitors.

In the same way, crucial advancements were made on the knowledge of the biochemistry, pharmacology and molecular biology of the PDE4 family, including a detailed characterization of the different PDE4 subtypes, differentially expressed in tissues and cells [66]. The identification of a variety of potent and selective compounds, defined as second-generation PDE4 inhibitors, sustained the hope that the lower emetic potential of these molecules should overcome the problems encountered with rolipram and its congeners, whose development as antiasthma drugs failed due to the adverse reactions [16]. This newly acquired information greatly stimulated the research leading to the synthesis of Roflumilast by AstraZeneca, which was finally approved as a COPD drug in the EU (in 2010) and in the USA (in 2011) [70]. Since the marketing of Roflumilast, three additional compounds have been marketed as PDE4-inhibitor drugs (Figure 4) i.e., Crisaborole (by Pfizer) for atopic dermatitis [71], Apremilast (by Celgene) for psoriatic arthritis [72] and Ibudilast (by MediciNova) for Krabbe diseases (also known as globoid cell leukodystrophy) [73]. However, research in the field of PDE4 inhibitors has remained very active, leading to very potent and pharmacologically relevant ligands that have entered clinical trials for the treatment of a range of diseases. This review aims to provide an overview of the most interesting compounds that have been developed and entered into clinical trials since the marketing of the first PDE4 inhibitor Roflumilast.

## 5. PDE4 Inhibitors under Development 

### 5.1. Asthma and COPD

Although asthma and Chronic Obstructive Pulmonary Disease (COPD) cannot be strictly classified as autoimmune diseases, the involvement of a variety of cells responsible for immune response and the upregulation of TNF-α in these respiratory disorders strongly suggests the benefit of drugs able to inhibit immunocompetent cell proliferation and cytokine production. Since the discovery of Rolipram as a potent and selective PDE4 inhibitor, asthma became the major therapeutic target for this agent and its congeners, as extensively covered by several excellent reviews [74,75,76,77,78].

COPD is a progressive lung disease affecting at least 6% of the population. In the USA, a 60% increase in prevalence was observed from 1982 to 1995 and COPD was the fourth leading cause of death in 2017 [79]. The definition COPD includes emphysema and chronic bronchitis which are both characterized by obstruction of the air flow. Most COPD cases (80–90%) are linked to smoking, although other causes are exposure to industrial pollutants and lung infections. In the last years, COPD has received less attention compared to asthma, as it has been considered an “intractable” air flow disorder, largely unresponsive to treatment with corticosteroids and α_2_-agonists, which are typically used in the treatment of asthma. However, COPD and asthma patients share similar clinical phenotypes, difficult to distinguish, particularly when they coexist. Currently, the therapies for COPD are mainly based on α_2_-agonists, anticholinergics, LTB_4_ antagonists and protease inhibitors [80,81,82,83], being the corticosteroids low effective/ineffective for the treatment of this neutrophilic inflammation. 

As a result, in the past decades, COPD became a major focus in the context PDE4 inhibitors, due to their ability to deeply suppress airway inflammation and relax smooth muscle via the elevation of cAMP levels. Research in this field culminated in the marketing of Roflumilast, as mentioned above, as an oral formulation for the treatment of severe COPD (EU and USA in 2010 and 2011, respectively). Due to the success of this new therapeutic approach, a huge effort was made by researchers to improve the potency, safety and tolerability of these drugs, by considering inhaled PDE4 inhibitors as a viable alternative [24]. In Table 1 are reported the molecules under development for the treatment of respiratory inflammatory diseases. Among compounds developed by Chiesi Farmaceutici [84,85,86], **CHF 6001** results the most promising and advanced compound in clinical trials. CHF 6001 is a potent PDE4 inhibitor that showed high preclinical efficacy [85] and it is also well-tolerated in humans [87]. In a double-blind study, 36 atopic asthmatics received the molecule (400 or 1200 µg inhaled once daily) for 9 days, resulting in a significantly inhibited allergen-induced late asthmatic response (for both doses) [88]. In a different study, 61 patients with COPD and chronic bronchitis received CHF 6001 at doses of 800 or 1600 µg inhaled twice daily for 32 days. Both doses of CHF 6001 significantly reduced multiple inflammation-related biomarkers demonstrating additional lung anti-inflammatory action [89]. Minimal side effects (gastrointestinal adverse effects comparable to placebo) were reported in the same study, demonstrating that CHF 6001 produces anti-inflammatory effects with high toleration by patients, compared to other PDE4 inhibitors. Currently, CHF 6001 is in phase IIb clinical trials for the treatment of COPD (Table 1). Another compound worthy of consideration is **GSK256066**, which is a potent PDE4 inhibitor (IC_50_ = 3.2 pM) developed by GSK [90]. In contrast to other PDE4 inhibitors, GSK256066 exhibited marked in vitro selectivity for PDE4 (vs. PDE1–7). GSK256066 has shown a protective effect on the early and late asthmatic responses in a randomized, double-blind study. Moreover, the molecule resulted well tolerated with limited systemic exposure when inhaled, obtaining good efficacies by minimizing side effects derived from systemic circulation of the molecule [90]. In a different study (phase IIa) GSK256066 was tested for a 4 week study using two different doses (25 and 87.5 mg). The results showed that there were no differences in the inflammatory markers between the two doses and the incidence rates of gastrointestinal side effects were negligible in all therapy groups [91,92]. **RPL554** or Ensifentrina is a mixed PDE4/PDE3 inhibitor endowed with a good efficacy and safety profile without cardiac side effects. Despite its low potency Ensifentrine causes bronchodilation and symptom improvements in COPD patients and it is under evaluation not as a first-line treatment, but rather as an adjuvant drug to existing long-acting bronchodilators for the treatments of COPD [93,94]. **Oglemilast** (GRC3886), by Forest Laboratories, is currently in phase II clinical trials for the treatment of COPD [95]. Finally, it is worth mentioning **Cilomilast** [96], **MK0873** [97] and **Revamilast** [98], the clinical trials of which have been discontinued from several years. After the commercialisation of Roflumilast, these compounds rapidly reached the clinical phase for the treatment COPD and asthma. In particular, Cilomilast was considered the most promising candidate, although the results were not sufficient to allow its marketing (Table 1).

### 5.2. Atopic Dermatitis (AD) and Psoriasis

Atopic dermatitis (AD) is a chronic inflammatory skin disorder that afflicts 2–3% of the population worldwide and its prevalence intensifies with increasing age. There is a high frequency of AD in subjects with a history of respiratory allergy [99]; moreover, 15% of the individuals afflicted by AD develop arthritis. The aetiology of this disorder, which is characterized by pruritis, cutaneous reactivity and erythema is unknown, but the implication of a variety of immune and inflammatory cell types strongly suggests a classification as an autoimmune disorder [100]. An important approach for the treatment of AD must be considered the use of moisturizers, necessary for the topical steroid preparations, the drugs of choice for this condition (hydrocortisone, betamethasone and clobetasol), but which have recently been joined by topical calcineurin inhibitors (TCIs) such as Tacrolimus (for adults only) and Pimecrolimus) [99,101].

Psoriasis is a chronic inflammatory disease that involves the skin and nails, the inflammation is manifested in the skin by localised or generalised patches and it is a lifelong condition [102]. For mild psoriasis, the main treatment is similar to that for the management of AD and it is represented by topical steroids, TCIs and vitamin D analogues [103]. Psoriasis can be associated with other comorbidities, including psoriatic arthritis (PsA) which sometimes occurs with joint inflammation and synovitis before the formation of plaques; PsA is a complication of psoriasis in 30% of people affected by the autoimmune disease. 

In the skin, PDE4 is primarily expressed in Langerhans cells, neutrophils, keratinocytes, and T cells, which also contribute to the psoriatic plaque formation and it has been demonstrated that PDE4 mRNA levels are higher in patients with psoriasis concerning healthy individuals [104]. For these evidences, PDE4 inhibitors have been investigated also in the field of skin disorders and psoriatic arthritis [100,105,106,107,108]. 

**Apremilast**, by Celgene [109], is the second approved PDE4 inhibitor in 2014 and was marketed for the treatment of psoriatic arthritis and moderate to severe plaque psoriasis that do not respond to topical glucocorticoid therapy. Previous preclinical study on arthritis and psoriasis in vivo models showed that the oral administration of Apremilast significantly mitigated the epidermal thickness and irregular proliferation and expression of ICAM-1, HLA-DR and TNF-α in the affected skin [105]. An in vivo study with BALB/c mice and DBA/1J mice confirmed the results, reducing the symptoms of arthritis with no measured side effects [58]. Some adverse effects have been reported in clinical trials for Apremilast such as headache, abdominal pain, depression, weight loss, nausea, diarrhoea, vomiting, nasopharyngitis, and upper respiratory tract infections [110]. However, the molecule offers an adequate therapeutic window and is well-tolerated in long-term exposure [108]. Apremilast is a well-tolerated drug and is currently in at least ten phase II and III clinical trials for different types of psoriasis, both for oral and topical use (Table 2). There are also two studies on paediatric subjects: the first one from 6 to 17 years of age with moderate to severe plaque psoriasis, the second with active juvenile psoriatic arthritis. Finally, Apremilast is also in development for Palmoplantar pustulosis and for nummular eczema.

**Crisaborole** (AN-2728, Eucrisa), the last licensed PDE4 inhibitor by Pfizer (December, 2016) [111] has been introduced as a drug for topical treatment of atopic dermatitis. Despite its moderate potency (IC_50_ = 490 nM), it shows excellent anti-inflammatory activity both in vitro and in vivo [112] and the animal toxicity studies indicated a wide safety margin in systemic and topical application [113,114]. Clinical trials of the molecule showed that treatment with 2% ointment twice daily was the most effective in relieving symptoms compared with the vehicle-controlled study [115,116]. Unlike systemic treatment, topical therapy of crisaborole failed to cause gastrointestinal adverse effects, probably because the molecule is rapidly metabolized into two inactive compounds after the topical application [117]. Crisaborole is actually in clinical trials to evaluate its antipsoriatic efficacy and safety at different concentrations of topical formulation in subjects with psoriasis vulgaris; it is also in development for different types of dermatitis, to compare the efficacy of Crisaborole with that of Hydrocortisone, Triamcinolone and Aquaphor (Table 2).

Other molecules currently under investigation with promising therapeutic efficacy for AD and psoriasis treatment are reported in Table 2. **E6005** (RVT-501) decreases pruritus and inflammation in both paediatric and adult AD patients without no relevant side effects when topically applied [118]. In a dose study, the skin lesion scores were reduced by the molecule in a dose-dependent manner [119]. **OPA-15406**, by Otsuka, is in a different clinical trial as a topical application for the treatment of AD, in particular in paediatric patients. The studied 1% ointment improved the pruritus score, without adverse effects in the 2 week study, assessing the efficacy and tolerability of topical treatment [120]. **Leo-29102** was developed by Leo Pharma in 2014 [121]. The molecules showed promising results for the treatment of AD patients resulting in a significant effects of the molecule on the assessment of pruritus and overall assessment of disease severity [20]. It recently also completed phase II clinical trials for Psoriasis vulgaris, as Leo 29102 cream, and in combination with calcipotriol and betamethasone. **Pefcalcitol** (M5181, Maruho Pharmaceutical) is a vitamin D3 analogue with PDE4 inhibitory activity. Preclinical studies showed that the molecule is an effective therapy for plaque psoriasis with fewer side effects than vitamin D3 when used in topical application [122]. It is actually in clinical trials for psoriasis in a multicentre study to assess the safety, tolerability and pharmacokinetic of 0.05% Pefcalcitol ointment in adolescent subjects. Additional new PDE4 inhibitors are in development for psoriasis, such as **Hemay005** [123], **Orismilast** [124] and **MK-0873** [125], while **GW842470X** [126] and **DRM02** for AD [127]; the latter have recently completed phase II as for AD and for rosacea (see Table 2).

### 5.3. Rheumatoid Arthritis and Lupus 

Rheumatoid arthritis (RA) is the most prevalent immune disorder, affecting 2.5 million people in the US with an estimated expense for therapy of more than 17 billion dollars each year [128]. RA can also affect young people, but more than 70% of the patients are over 30 years old [129]. Mortality rates in RA are increased at least two-fold and are linked to clinical severity [130]. In RA the joint lining swells, invading the surrounding tissues and producing proteases that attack and destroy the joint surface. Although this generally occurs in feet and hands, larger joints such as hips, knees and elbows also may be involved. Swelling, pain and stiffness are usually the main symptoms, even when the subject is at rest. There are well-defined protocols in the treatment of AR taking into account numerous factors, in particular, the development stage of the disease; the drugs used in the first phase of the pathology or pending a precise diagnosis are NSAIDs and glucocorticoids, while Disease-Modifying Anti-Rheumatic Drugs DMARDS are used after certain diagnosis and with the fully developed disease. Of the latter, the drugs of the first choice are conventional synthetic DMARDs, of which Methotrexate is the most widely used, followed by the newer Biologic DMARDs and Targeted Synthetic DMARDs [131,132,133,134].

Given the high costs and severe side effects of these drugs, research has turned to other targets, including PDE4 [135]. The rational basis for the possible utility of PDE4 inhibitors in RA is the evidence that the c-AMP increase in leukocytes is associated with the block of several leukocyte functions, including TNF-α production and the subsequent release of other inflammatory mediators and reactive oxygen species [136]. This approach is largely justified since some biologic DMARDs actually in use, such as Etanercept and Infliximab, act precisely as TNF-α inhibitors. TNF-α plays a pathogenic role in the establishment of rheumatoid synovitis, in the formation of pannus tissue and joint destruction by increasing synoviocyte proliferation and triggering a cascade of secondary mediators which are involved in neo-angiogenesis, recruitment of inflammatory cells and in the final joint destruction [137]. The first evidence of this possible therapeutic application emerged during a study in the 1990s, carried out with PDE4 inhibitors, including rolipram, aimed to demonstrate modulation of TNFa by these compounds [138]. Further studies performed on Roflumilast, which had just come onto the market for COPD [139], and also on Apremilast [58], which would not be approved for psoriatic arthritis until some years later, highlighted the potential of these compounds for RA demonstrating a direct correlation between PDE4 inhibition and synovial TNF-α and inflammatory cytokine and chemokine release in human synovial cells. Moreover, Apremilast reduced in a murine model of RA the degeneration of tibiotarsal joint in a dose-dependent manner. Other in vivo studies demonstrated that weak and non-specific PDE inhibitors such as theophylline significantly reduced oedema in rat adjuvant arthritis models [140]. Also Ibudilast, after its commercialisation for Krabbe’s disease in 2016, has been tested in vitro and mouse models of RA [141] and it is able not only to reduce the expression and secretion of inflammatory mediators but also to inhibit the disease progression. Given its established safety profile, Ibudilast is a good candidate to enter clinical trials for RA.

In the past decade, three compounds have entered clinical trials for RA: **Revamilast** (GRC 4039, Glenmark) [142], **MK0873** by Merk [125] and the drug **Apremilast** (Table 3). Revamilast was under evaluation for different inflammatory disorders, and it finished phase I for RA treatment, showing a significant TNF-α inhibition in healthy human volunteers [16]. It also completed a phase IIb study to determine the safety and tolerability in patients with active rheumatoid arthritis who have shown an inadequate response to Methotrexate, but the results are not published yet (ClinicalTrials.gov Identifier: NCT01430507). The clinical trial of MK0873 was completed in 2014, while phase II of Apremilast was discontinued due to lack of efficacy.

**Figure 5:**
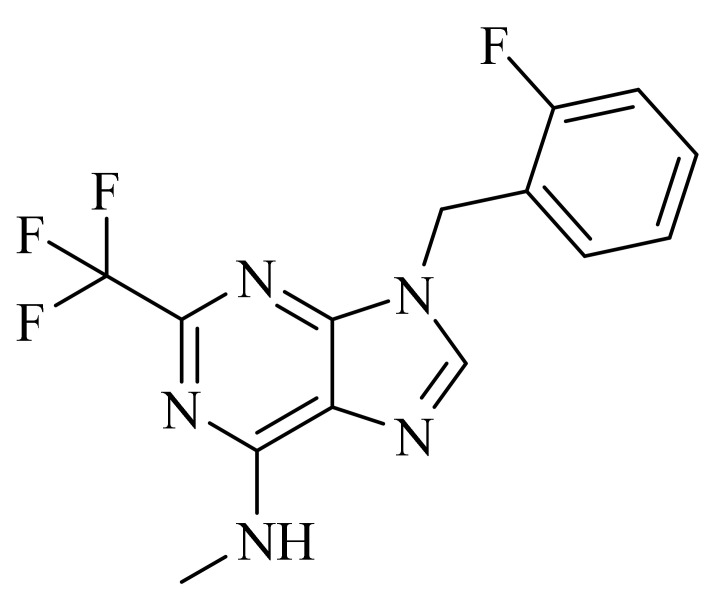
Structure of compound NCS613 (preclinical study for Lupus).

Systemic Lupus Erythematosus (SLE) is a complex autoimmune multisystemic disease characterized by erythematous skin, mucosal manifestations, and systemic involvement of almost all organs and apparatuses such as the kidney, joints and central nervous system. Unlike rheumatoid arthritis, lupus is less disabling and usually does not cause severe destruction of the joints [143]. Current therapy for the treatment of SLE involves the use of FANS, antimalarials, corticosteroids, and DMARDs such as Methotrexate and Rituximab [144,145]. Due to positive anti-inflammatory properties, PDE4 inhibitors have been also proposed as an interesting alternative/complementary choice for the management of SLE [146]. Compound **NCS613** (Figure 5) has been extensively studied and it demonstrated to be able to reduce proteinuria and increase the survival rate in MRL/lpr lupus-prone mice showed that NCS613. The molecule also inhibited basal and LPS-induced TNFα secretion from PBMCs of lupus patients, which was also noted in the mice as well [147]. In a different study, NCS613 was reported to downregulate PDE4B and, meanwhile, upregulated PDE4C expression in healthy humans and in lupus patients. Moreover, NCS613 reduced the level of TNF-α, IL-6, and IL-8 due primarily to the abolishment of phosphorylation of p38 MAPK and the translocation of NF-κB [148]. A recent study showed that NCS613 suppresses the immune complex deposition of MRL/lpr lupus-prone mice in the kidney, and since nephritis is the most common and severe manifestation of SLE, this compound could be a leading drug candidate for this pathology [149].

Finally, Apremilast has also entered clinical trials for Discoid Lupus Erythematosus and phase II has been completed in 2021 (Table 3). No published data are available.

### 5.4. Neurological Disorders

Multiple sclerosis (MS) is a chronic inflammatory autoimmune and neurodegenerative disease affecting the central nervous system (CNS) and a typical marker of this disorder is the presence in CNS of sclerotic plaques characterized by demyelination which reduces stimulus conduction, provoking a progressive physical impairment [150,151]. The factors contributing to the onset and progression of this disease are many and complex, but genetic and environmental ones are certainly the most important. About 350,000 people in US suffer for MS, which, together with trauma, is the top cause of disability in young adults. In the early stages of the disease, most of the patients experiments relapsing and remitting periods. Symptoms vary greatly and depend on the location of lesions in the CNS, but generally include problems with vision and touch, muscle weakness, difficult bladder control and sexual dysfunction. Impairment of memory, attention and concentration generally affects up to 60% of patients: in the first stage of the disease, symptoms can go into remission for a long time, but suddenly severe attacks turn up again. In other cases, symptoms worsen gradually over the years. Cognitive problems are a big part of the disease for a lot of patients [150]. Currently, the first-line therapies for AR are immunomodulatory or immunosuppressive therapies and among the used drugs are Interferon-β 1a and 1b (IFN-β1a and 1b), Glatiramer acetate, Teriflunomide, Fingolimod, Natalizumab and Mitoxantrone, the most of these compounds showing severe adverse effects [150]. Since inflammation is a fundamental component of neuroinflammatory and neurodegenerative responses in MS, the anti-inflammatory properties of PDE inhibitors can be an attractive choice to impede peripheral lymphocyte accumulation by reinforcing the BBB, as well as to restore the balance between pro- and anti-inflammatory mediators [152].

The experimental evidence of PDE4 inhibitors efficacy against MS dates back to the 1990s when Rolipram was tested in a model of experimental allergic encephalomyelitis (EAE), an animal model generally accepted for the study of MS. Rolipram administered sc every 48 h for 45 days at 10 mg/Kg completely suppressed the clinical signs of EAE in marmosets [153] confirmed by magnetic resonance imaging of the marmosets brain, that showed the disappearance of abnormalities in comparison with vehicle-treated animals. Moreover, Genain et al. [154] found that brain TNF mRNA levels were lower in Rolipram-treated marmosets that showed no clinical signals of EAE. Unfortunately, these very exciting results were not followed by the same success in clinical studies because of the presence of severe adverse events. Other than common nausea, vomiting and insomnia, the patients treated with Rolipram reported an increase in the amount of contrast-enhanced lesions (CEL) compared to the baseline state [155].

At the present, only the drug **Ibudilast** completed the phase II clinical trials for Multiple Sclerosis and results seem to indicate its effect on brain atrophy, perhaps associated with slower disease progression. However, this drug shows the usual side effects of PDE4 inhibitors, such as gastrointestinal symptoms and headache [156]. The same drug is in three different studies for another form of sclerosis, Amyotrophic Lateral Sclerosis (ALS), also known as motor neuron disease, a pathology characterized by the degeneration of both upper and lower motor neurons [157]. It is a progressive and fatal neurodegenerative pathology in which there is a progressive weakness of the voluntary muscles and muscles of breathing, swallowing and speech [158]. Ibudilast has already completed phase II of two different clinical trials, the first in which it was used alone, the second in association with Riluzole, the only drug currently available for ALS. A further study started in June this year and is currently being recruited (Table 4). 

PDE4-targeted therapy has also shown promising results in other neurological disorders and the same Rolipram, the first blood-brain-barrier permeable PDE4 inhibitor, has been proven to be effective in various animal models of Parkinson’s disease, Alzheimer’s disease, depression and neuropathic pain [159,160,161]. Currently, there are some PDE4 inhibitors in clinical trials for different neurological disorders (Table 4) such as Alzheimer’s disease (AD), fragile X syndrome, depression and Huntington’s disease [23]. As well known, AD is a neurodegenerative pathology characterized by the deposition of amyloid beta-peptide (AB) (so-called senile plaques) and tau protein in the form of neurofibrillary tangles. The involvement of cAMP and cGMP on AB production has long been known [162], as well as the increase in PDE4 expression in the early stages of AD [163], thus indicating the interest in PDE4 inhibitors for the treatment of this pathology [164]. Two promising compounds, **MK0952** and **BPN14770**, as well as the drug Roflumilast, have been developed for the treatment of AD and reached phase 2 and 1 clinical trials, respectively. BPN14770 in particular has participated in several clinical trials, the last of which (NCT03817684) is still active in phase 2. 

It has long been known that in depressive patients there is a dysfunction of the AMPc signalling pathway [165,166], and it was shown that the use of PDE4 inhibitors, such as Rolipram, enhances the antidepressant effect when used in combination with beta1 or beta2 agonists in mouse models [167]. By increasing cAMP levels, depression has also become an attractive target for PDE4 inhibitors [168] and in this last decade, three compounds have entered clinical trials: **BPN14770**, which completed Phase II in 2021, **GSK356278** and **Roflumilast** whose estimated primary completion date is October 2022. GSK356278 has also completed two phase I clinical studies (one alone and one in addition to Rolipram) in 2017, for the treatment of Huntington’s disease (HD), an autosomal, progressive neurodegenerative disease characterized by a progressive motor and cognitive decline with a sorrowful prognosis [169]. 

A recently identified target for PDE4 inhibitors is fragile X syndrome, a genetic disease caused by the alteration of FMR1 gene [170]. It represents the second cause of intellectual disability and it shows itself with autistic-like behaviour and hyperactivity. This pathology is strictly associated with reduced cAMP levels in mice and the use of **BPN14770**, a potent PDE4D allosteric inhibitor [171] in the animal model of FXS (Fmr1 KO), afforded positive effects, such as hyperactivity decrease and behaviour improvement [172]. BPN14770 completed phase II clinical trials in 2020 and the results are encouraging, since the treatment of the patients with an oral dose 25 mg twice daily affords significant cognitive improvement, together with good tolerability [173]. It is now entering phase III with three different groups of patients, divided by age (Table 4).

Finally, [11C](R)-rolipram showing good properties such as high affinity (IC_50_ = 1–2 nM) and lipophilicity (Log P about 3) is an interesting positron emission tomography (PET) brain imaging agent. For these characteristics, **Rolipram** has entered clinical trials to measure PDE4 levels in the brain of patients with various neurological and psychiatric disorders (NCT00369798) to evaluate the different levels of PDE4 in patients with McCune-Albright Syndrome (MAS) compared to healthy controls (NCT02743377) (Table 4).

### 5.5. COVID-19 and PDE4

Recently, because of the emergency related to the SARS-CoV-2 pandemic, interest has arisen in the use of PDE4 inhibitors in the treatment of severe symptoms characterizing COVID-19, both for their anti-inflammatory and antiviral effect on HIV-1 replication [174]. As known, COVID-19 disease is characterized by a hyperinflammatory state due to a massive release of pro-inflammatory cytokines, called a “cytokines storm”, and effective inhibitory action on cytokines is also performed by cAMP, through modulation of other protein pathways. Thus, PDE4 could represent an attractive new target for the development of potential drugs and the use of PDE4 inhibitors could prevent the storm of cytokine responsible for the major compliances of COVID-19 [175,176]. Of interest, phase 3 clinical trials have been completed (November 2021) for the drug Apremilast (previously in the market as anti-psoriasis agent) for the treatment of SARS-CoV-2 infection (ClinicalTrials.gov Identifier: NCT04590586). 

## 6. Conclusions

Increasing the intracellular cAMP levels by PDE4 inhibition has been proven to be an effective strategy for the treatment of several inflammation diseases and after more than twenty years from the pioneering studies of Crummey, Thorphy and coworkers suggesting the potential of in asthma therapy, different molecules PDE4 inhibitors have been marketed. Since Roflumilast reached the market, the enormous therapeutic potential for PDE4 inhibitors in various diseases has been studied and several clinical trials covering different aspects of PDE4 inhibitions in several diseases have been reported. Among the whole plethora of PDE4 inhibitors, Roflumilast was initially approved for the treatment of COPD and asthma, apremilast for the treatment of psoriasis and psoriatic arthritis and crisaborole was approved for the treatment of AD.

In addition to these applications, the currently know PDE4 inhibitors are being tested for other different inflammation based diseases. Recent applications of PDE 4 inhibitors also include the treatment of Behcet’s syndrome, where apremilast is being applied and for the treatment of hepatic steatosis unassociated with alcohol, where ASP9831, developed by Astellas Pharma Inc., exhibited potent anti-inflammatory and antifibrotic effects in preclinical studies. The aim of this review was to provide the reader with an overview of PDE4 targeting compounds that have reached clinical trials in the last ten years, since the first PDE4 inhibitor was marketed, with a focus on those most recently developed for respiratory, skin and neurological disorders. Unfortunately, despite that small molecule PDE4 inhibitors have been widely studied as therapeutics for several human inflamatory-based diseases, only a few of them have been able to be approved, due to side effects as PDE4 is widely expressed in many tissues. Therefore, one problem that remains to be solved is the achievement of tissue- and cell-specificity for specific therapeutic aims. It appears evident that the research in this field is an avenue worth purchasing, but an analysis of the pharmacological profile of classical PDE 4 inhibitors, clearly indicated that there are several charateristics to be improved for novel drug candidates of this class such as nanomolar activity at the catalytic site of PDE 4, low affinity for HARBS, selectivity for the PDE subtype, potent TNF-*α* inhibitory activity, low brain penetration, combination therapy, change the routine of administration, (inhaled administration of GSK256066, that limited the systemic exposure with less to none gastrointestinal side effects, and topical application crisaborole did not cause any gastrointestinal adverse effects).

Although there are still drawbacks, the several compounds that are currently in clinical trials and data reported in this review strongly encourage researchers to chase the therapeutic the potential of PDE4 inhibitors in order to propose novel therapeutic for human diseases.

## Figures and Tables

**Table 1:** PDE4 inhibitors under development for the treatment of respiratory inflammatory diseases.

Compound	Chemical Structure	Company	Phase	NCT Number ^a^
**CHF6001** **(Tanimilast)**	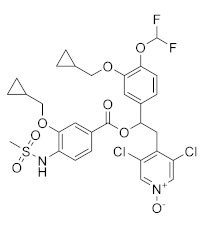	Chiesi Farmaceutici	Phase III (COPD)	NCT04636801 (2022) NCT04636814 (2021)
			Phase II (Asthma)	NCT01689571 (2017)
			Phase II (COPD)	NCT03004417 (2020) NCT01730404 (2017) NCT02986321 (2019)
			Phase I (COPD)	NCT04756960 (2021) NCT05373953 (2022) NCT04739774 (2021) NCT02386761 (2020) NCT05431426 (2022) NCT01703052 (2020)
**GSK256066**	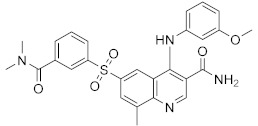	GSK	Phase II (Asthma)	NCT00549744 (2017)
			Phase II (COPD)	NCT00549679 (2017) NCT00515268 (2017)
**RPL554**	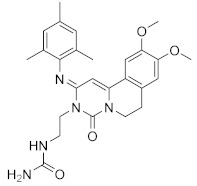	Verona Pharma	Phase II (COPD)	NCT04027439 (2021) NCT03937479 (2020) NCT03673670 (2019) NCT03443414 (2019) NCT04091360 (2021) NCT05270525 (2022)
**Oglemilast**	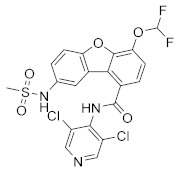	Forest Laboratories	Phase II (COPD)	NCT00671073 (2019)
**Cilomilast**	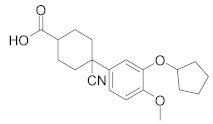	GlaxoSmithKline	Phase III (COPD)	NCT00103922 (2016)
**MK0873**	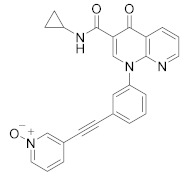	Merck Sharp & Dohme LLC	Phase II (COPD)	NCT00132730 (2018)
**Revamilast** **(GRC 4039)**	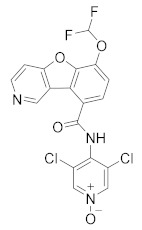	Glenmark Pharmaceuticals Ltd. India	Phase II (Asthma)	NCT01436890 (2013)

^a^ ClinicalTrials.gov identifiers from https://clinicaltrials.gov (last accessed on July 14 2022).

**Table 2:** PDE4 inhibitors under development for the treatment of atopic dermatitis (AD) and psoriasis.

Compound	Chemical Structure	Company	Phase	NCT Number ^a^
**E6005**	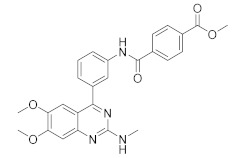	Dermavant Sciences	Phase II (AD)	NCT01461941 (2018)
			Phase I/II (AD)	NCT01179880 (2018) NCT02094235 (2018)
**GW842470X**	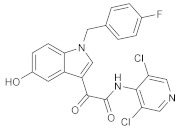	GSK	Phase II (AD)	NCT00354510 (2012)
			Phase I (AD)	NCT00356642 (2017)
**OPA-15406**	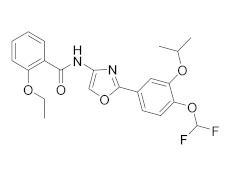	Otsuka	Phase III (AD)	NCT05372653 (2022) NCT03961529 (2021) NCT03908970 (2021) NCT03911401 (2021)
			Phase II (AD)	NCT02068352 (2021) NCT02914548 (2020) NCT03018691 (2020) NCT02945657 (2018)
			Phase I (AD)	NCT02334787 (2016) NCT01702181 (2014)
**Leo-29102**	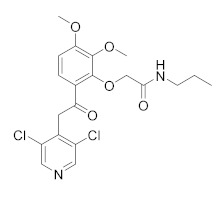	Leo Pharma	Phase II (AD)	NCT01037881 (2019)
			Phase II (Psoriasis Vulgaris)	NCT00875277 (2019)
			Phase I (AD)	NCT00891709 (2016) NCT01447758 (2013) NCT01005823 (2013) NCT00958516 (2013) NCT01423656 (2013)
			Phase I (Psoriasis Vulgaris)	NCT01466478 (2013)
**DRM02**	Undisclosed	Dermira	Phase II (Rosacea)	NCT01993446 (2021)
			Phase II (AD)	NCT01993420 (2021)
			Phase II (Psoriasis)	NCT01993433 (2021)
**Pefcalcitol** **(M518101)**	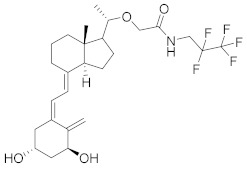	Maruho	Phase III (Psoriasis)	NCT01908595 (2015)
			Phase II (Psoriasis)	NCT02970331 (2019)
**Hemay005**	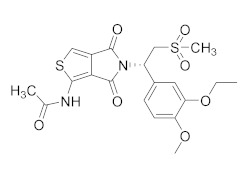	Tianjin Hemay Pharmaceutical Co., Ltd.	Phase III (Psoriasis)	NCT04839328 (2022)
			Phase II (Psoriasis)	NCT04102241 (2021)
			Phase I (Psoriasis)	NCT04837235 (2021) NCT03007810 (2018) NCT03570346 (2018)
**Orismilast** **(LEO-32731)**	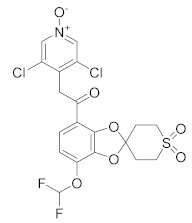	UNION therapeutics	Phase II (Psoriasis and Skin Diseases)	NCT05190419 (2022)
**MK-0873**	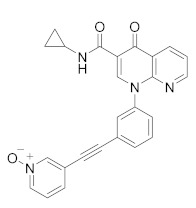	Merck Sharp & Dohme LLC	Phase I (Psoriasis Plaque)	NCT01140061 (2019) NCT01235728 (2019)
**Apremilast** **(CC-10004)**	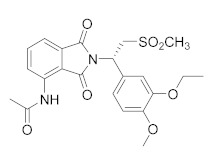	Amgen	Phase IV (Psoriasis)	NCT03022617 (2021) NCT02400749 (2018)
			Phase III (different type of Psoriasis)	NCT03777436 (2022) NCT04175613 (2022) NCT01194219 (2022) NCT03701763 (2022) NCT03930186 (2022) NCT05174065 (2022) NCT04804553 (2022) NCT03721172 (2021)
			Phase II (Psoriasis)	NCT04572997 (2021) NCT00604682 (2020) NCT00606450 (2020)
			Phase II (AD and eczema)	NCT04306965 (2021) NCT03160248 (2021)
**Crisaborole (AN2728)**	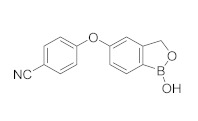	Pzifer	Phase IV (AD and eczema)	NCT03832010 (2022) NCT04023084 (2021)
			Phase IV (Seborrheic Dermatitis)	NCT03567980 (2021)
			Phase II (AD and eczema)	NCT04091087 (2022) NCT01652885 (2017)
			Phase II (Morphea)	NCT03351114 (2021)
			Phase II (Psoriasis)	NCT00759161 (2017) NCT01300052 (2017) NCT00759161 (2017) NCT01029405 (2017)
			Phase I (Psoriasis)	NCT00762658 (2019) NCT00763204 (2019)

^a^ ClinicalTrials.gov identifiers from https://clinicaltrials.gov (last accessed on July 14 2022).

**Table 3:** PDE4 inhibitors under development for the treatment of rheumatoid arthritis (RA) and lupus.

Compound	Chemical Structure	Company	Phase	NCT Number ^a^
**Revamilast** **(GRC 4039)**	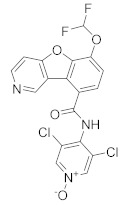	Glenmark Pharmaceuticals Ltd. India	Phase II (RA)	NCT01430507 (2012)
**MK0873**	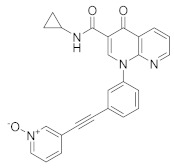	Merck Sharp & Dohme LLC	Phase II (RA)	NCT00132769 (2015)
**Apremilast** **(CC-10004)**	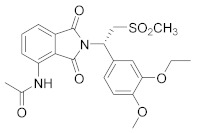	Amgen	Phase II (RA)	NCT01285310 (2020) NCT01250548 (2014)
			Phase I/II (lupus)	NCT00708916 (2021)

^a^ ClinicalTrials.gov identifiers from https://clinicaltrials.gov (last accessed on July 14 2022).

**Table 4:** PDE4 inhibitors under development for the treatment of different neurological disorders.

Compound	Chemical Structure	Company	Phase	NCT Number ^a^
**Ibudilast** **(MN-166)**	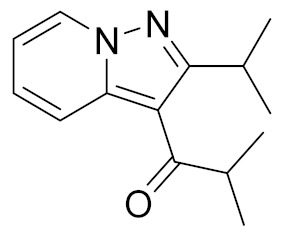	Medicinova	Phase II (ASL)	NCT04057898 (2022) NCT02714036 (2020) NCT02238626 (2021) NCT01982942 (2020)
			Phase II (Glioblastoma)	NCT03782415 (2022)
			Phase I (Migraine Headache)	NCT01389193 (2015)
**BPN14770** **(Zatomilast)**	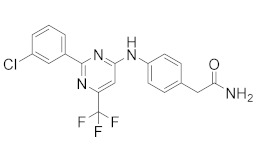	Shionogi Pharma, Tetra Therapeutics	Phase III (Fragile X Syndrome)	NCT05367960 (2022) NCT05358886 (2022) NCT05163808 (2022)
			Phase II (Fragile X Syndrome)	NCT03569631 (2020)
			Phase II (Alzheimer disease)	NCT03817684 (2019)
			Phase II (Depression)	NCT03861000 (2021)
			Phase I (Alzheimer disease)	NCT02648672 (2017) NCT02840279 (2017) NCT03030105 (2018)
**GSK356278**	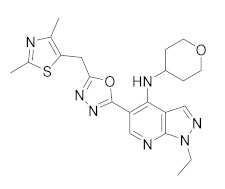	GlaxoSmith Kline	Phase I (Huntington disease)	NCT01602900 (2017) NCT01573819 (2017)
			Phase I (Depressive Disorder and Anxiety Disorders)	NCT01031186 (2017)
**MK-0952**	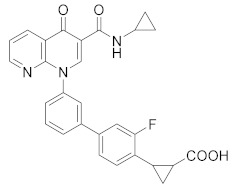	Merck Sharp & Dohme LLC	Phase II (Alzheimer disease)	NCT00362024 (2016)
**Roflumilast**	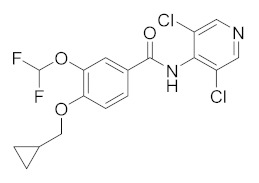	AstraZeneca	Phase II (Dementia)	NCT04658654 (2022) NCT01433666 (2020)
			Phase II (Cerebrovascular Disorder)	NCT04854811 (2021)
			Phase II (Nervous System Disease)	NCT02743377 (2020)
			Phase I (Major Depressive Disorder)	NCT04751071 (2022)
			Phase I (Schizophrenia)	NCT02079844 (2016)
			Phase I (Memory ImpairmentAlzheimer’s Disease)	NCT02051335 (2017)
			Phase I (Fragile X Syndrome)	NCT05418049 (2022)
**Rolipram**	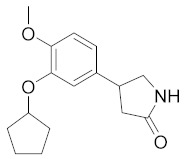	Schering AG	Phase II (Nervous System Disease, PET)	NCT02743377 (2020)
			Phase I (Major Depressive Disorder, PET)	NCT00369798 (2018)

^a^ ClinicalTrials.gov identifiers from https://clinicaltrials.gov (last accessed on July 14 2022).

## Data Availability

Data available in a publicly accessible repository.
